# Both the intratumoral immune and microbial microenvironment are linked to recurrence in human colon cancer: results from a prospective, multicenter nodal ultrastaging trial

**DOI:** 10.18632/oncotarget.25276

**Published:** 2018-05-04

**Authors:** Juliana Noguti, Alfred A. Chan, Bradley Bandera, Colin J. Brislawn, Mladjan Protic, Myung S. Sim, Janet K. Jansson, Anton J. Bilchik, Delphine J. Lee

**Affiliations:** ^1^ Dirks/Dougherty Laboratory for Cancer Research, Department of Translational Immunology, John Wayne Cancer Institute, Providence Saint John's Health Center, Santa Monica, CA, USA; ^2^ Los Angeles Biomedical Research Institute, Harbor – UCLA Medical Center, Torrance, CA, USA; ^3^ Department of Surgical Oncology. The John Wayne Cancer Institute at Providence St. John's Health Center, Santa Monica, CA, USA; ^4^ Earth and Biological Sciences Directorate, Pacific Northwest National Laboratory, Richland, Washington, USA; ^5^ University of Novi Sad, Faculty of Medicine, Novi Sad, Serbia; ^6^ Oncology Institute of Vojvodina, Sremska Kamenica, Serbia; ^7^ UCLA Department of Medicine, Statistics Core, Los Angeles, CA, USA; ^8^ Division of Dermatology, Department of Medicine, Harbor - UCLA Medical Center, Torrance, CA, USA; ^9^ David Geffen School of Medicine, University of California - Los Angeles, Los Angeles, CA, USA

**Keywords:** colon cancer, microbiota, immune infiltrate, disease free survival, immune cells

## Abstract

Colon cancer (CC) is the third most common cancer diagnosed in the United States and the incidence has been rising among young adults. We and others have shown a relationship between the immune infiltrate and prognosis, with improved disease-free survival (DFS) being associated with a higher expression of CD8^+^ T cells. We hypothesized that a microbial signature might be associated with intratumoral immune cells as well as DFS. We found that the relative abundance of one Operational Taxonomic Unit (OTU), OTU_104, was significantly associated with recurrence even after applying false discovery correction (HR 1.21, CI 1.08 to 1.36). The final multivariable model showed that DFS was influenced by three parameters: N-stage, CD8^+^ labeling, as well as this OTU_104 belonging to the order Clostridiales. Not only were CD8^+^ labeling and OTU_104 significant contributors in the final DFS model, but they were also inversely correlated to each other (p=0.022). Interestingly, CD8^+^ was also significantly associated with the microbiota composition in the tumor: CD8^+^ T cells was inversely correlated with alpha diversity (p=0.027) and significantly associated with the beta diversity. This study is the first to demonstrate an association among the intratumoral microbiome, CD8^+^ T cells, and recurrence in CC. An increased relative abundance of a specific OTU_104 was inversely associated with CD8^+^ T cells and directly associated with CC recurrence. The link between this microbe, CD8^+^ T cells, and DFS has not been previously shown.

## INTRODUCTION

According to the American Cancer Society, colorectal cancer is the third most common cancer diagnosed in both women and men in the United States [1]; comprising a disease mortality rate of nearly 33% in the developed countries [2]. Although the median age at diagnosis is 66 years for males and 70 years for females [3], the incidence and mortality have been rising among young adults [4]. While many cancers have specific etiologic factors, no single risk factor accounts for most cases of colorectal cancer [5]. Major risk factors described include age, male gender, family history of previous cancer, inflammatory bowel disease, smoking, alcohol consumption, and high consumption of red meat and processed food [5–8].

Our group has also demonstrated in prospective international multicenter trials that adherence to surgical (the removal of ≥ 12 lymph nodes – LNs) and pathological (ultra-staging of LNs) quality measures significantly correlate with DFS in colon cancer (CC). This work was the first to clearly underscore the significant impact that these measures have on disease staging and outcome in CC, and emphasize the importance of adherence to surgical and pathological quality measures for any study seeking to evaluate new prognostic indicators to stratify patients with CC [9].

In addition, emerging evidence indicates an association of bacteria with gastrointestinal cancers [10]. With the advent of sequencing methodologies, investigators have identified nonculturable microbial genomes [11], leading to the discovery of a complex system composed of microbes inhabiting human surfaces and organs [12]. Specifically, several studies have investigated the association of bacteria in the development of CC [13–19]. A complex system composed of varied microbes, mucosal surfaces, and immune cells are regulated by other factors such as diet [20] and medication [21], creating a unique environment contributing to normal physiology and pathology [22]. However, when the gut microbiota is altered [23], this may lead to a pro-inflammatory environment [10, 24], favoring development and progression of CC [25, 26]. Inflammation can create conditions that alter the abundance of various resident bacteria which modify mucosal permeability. Together these changes may lead to translocation of other microbial species, toxins, inflammatory mediators, and immune cells [27, 28].

Recently cytotoxic and memory T cells infiltrating and surrounding the tumor were demonstrated to be more efficient than TNM stage classification by AJCC for predicting CC patient outcomes [29–33]. Consistent with these studies, we have previously shown that higher expression of CD8^+^ levels in the tumor center and invasive margin was associated with improved DFS in CC [34]. Pages et al. showed that high levels of intratumoral memory T cells were associated with decreased tumor recurrence and a better patient survival [29, 33]. Given the relationship between immune responses, microbiota, and their potential interactions to influence the surrounding host tissue, CC, by the very nature of its anatomic location, places the cancer cells in close contact with both elements. Therefore, we hypothesized that the gut microbiota within the tumor microenvironment may be associated with the immune response and recurrence in CC.

## RESULTS

### The microbiota of colon cancer tissue

**Figure 1:**
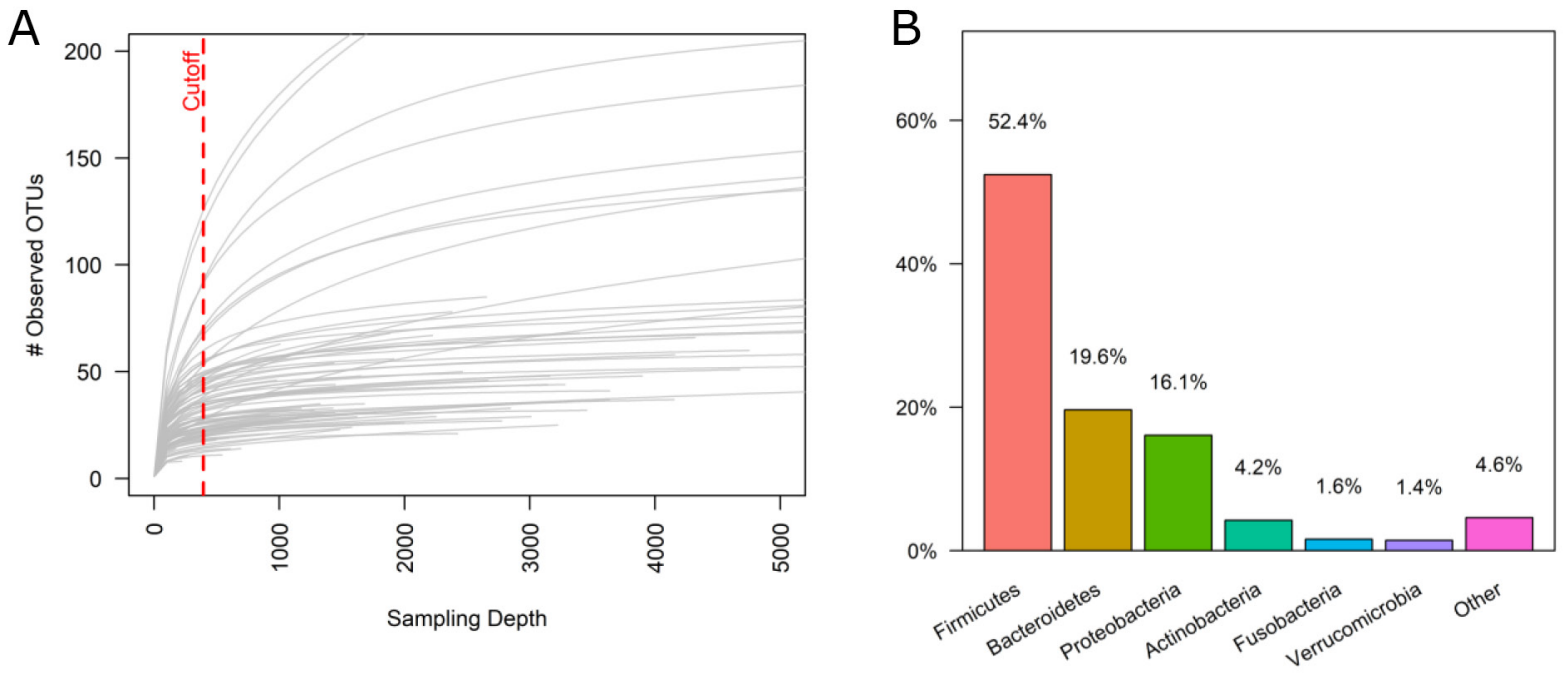
16S microbiome summary from FFPE colon tissue **(A)** Rarefaction curve showing the number of unique OTUs observed over the number of reads sampled. Each line represents one specimen from the dataset. Data table was rarefied to a sampling depth of 394 reads as indicated by the dashed red line. Six samples did not have sufficient quality reads and were omitted from downstream analysis. **(B)** Bar graph showing the average phylum-level distribution amongst the colon cancer tissue samples.

We investigated the microbiota from 91 FFPE colon cancer tissue samples, randomly selected from the only prospective clinic trial evaluating staging in colon cancer with attention to both surgical and quality standards. The median read per sample was 13,621. After subtracting OTUs found in the negative controls, the median read per sample was 1,915. Six samples were removed due to insufficient number of high quality reads and the 85 remaining samples were rarefied to sampling depth of 394 reads. The rarefaction curve is a plot of the number of unique OTUs over the number of reads sampled (Figure 1A). All of the specimens were sampled past their initial rapid increase in the number of OTUs observed, and most of the samples reached a plateau, indicating that the sampling depth of 394 reads provided sufficient coverage to capture a representative bacterial community without oversampling sequencing artifacts. We found a total of 972 operational taxonomic units (OTUs) with a table density of 0.037. The most abundant bacteria were those belonging to the phylum Firmicutes (52.4%), Bacteroidetes (19.6%), Proteobacteria (16.1%), and Actinobacteria (4.2%) (Figure 1B).

### DFS with clinical features, T cell markers, and tumor tissue microbiome

To determine the candidate variables associated with DFS, we first evaluated clinical, immunological [34], and microbiome-related data by univariable cox regression (Supplementary Table 1). Clinical, immunologic, and microbial features meeting the alpha threshold of 0.20 were included in the initial model for multivariable DFS analysis. Among the clinical parameters, age, AJCC stage, N-stage, and whether lymph nodes were affected met this threshold and were included in the initial model. Among the immunologic markers, CD3, CD4, and CD8 stains were included. Lastly, among the microbiome-related parameters included the three OTUs found in Table 1.

**Table 1:** Disease free survival and the intratumoral tissue microbiome

OTU	pval	p.adj	Hazard Ratio (95% CI)	Order	Family	Genus	Species
**OTU_104**	**0.001**	**0.036**	**1.21 (1.08, 1.36)**	**Clostridiales**	**NA**	**NA**	**NA**
OTU_213	0.049	0.882	1.21 (1.00, 1.46)	Actinomycetales	Corynebacteriaceae	*Corynebacterium*	NA
OTU_139	0.163	0.914	1.12 (0.94, 1.34)	Clostridiales	Lachnospiraceae	NA	NA

The table shows results from cox proportional hazard regression at the OTU-level under an alpha threshold of 0.20. Geographic location was added as covariate to the DFS model to account for batch effect between the two cohorts. The text in bold points out OTU_104, which was statistically significant after adjusting for multiple hypothesis testing.

**Figure 2:**
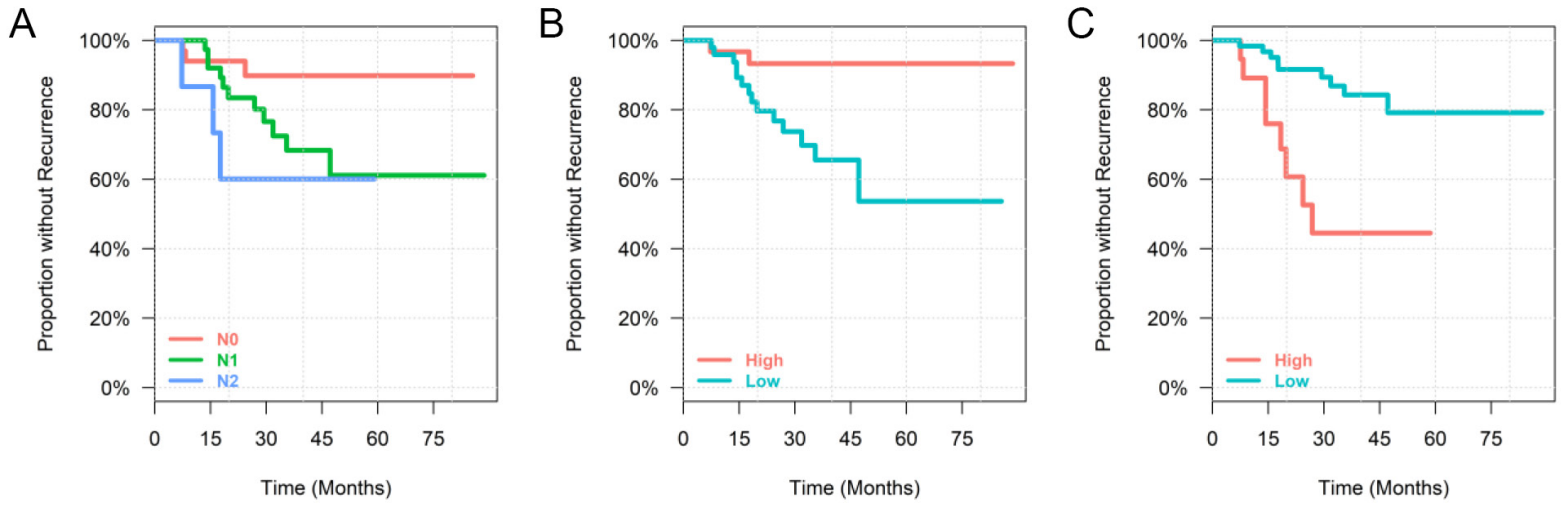
Kaplan–Meier plots for each of the predictor variables in the final DFS cox regression model Continuous variables are split into “low” and “high” group by the mean for easier interpretation. **(A)** Time until recurrence by N-Stage, **(B)** Time until recurrence by CD8, **(C)** Time until recurrence by OTU_104.

The final multivariable DFS model included N-Stage (Lymph Nodes Involved), CD8^+^ stain, and OTU_104 (Table 2). Patients with N-Stage 2 were 13.82 (CI: 2.52, 75.81) times more likely to have a recurrence than patients with N-Stage 0. For each unit increase of CD8^+^ stain, there is a 64% (CI: 38%, 79%) less chance of the tumor recurring. Lastly, for each unit increase in relative abundance of OTU_104, there is a 1.21 (CI: 1.05, 1.39) times more chance of recurrence. Each of the parameters in the final model are shown in Kaplan–Meier plots (Figure 2).

**Table 2:** Multivariable cox regression model on disease free survival

	Hazard Ratio	(95% CI)	P-Value
**N-Stage: N1 vs N0**	1.68	(0.43, 6.56)	0.453
**N-Stage: N2 vs N0**	13.82	(2.52, 75.81)	0.002
**CD8**	0.36	(0.21, 0.62)	< 0.001
**OTU_104**	1.21	(1.05, 1.39)	0.005

n = 84 (1 sample omitted for missingness). events = 16.

The final multivariable DFS model included N-Stage (Lymph Nodes Involved), CD8^+^, and OTU_104.

### Colon cancer microbiome in relation to CD8^+^ stain and N-Stage

Given our results that DFS is significantly associated with OTU_104, the CD8^+^ marker, and N-Stage, we hypothesized that the bacterial composition is also associated with CD8^+^ levels and/or with stage. The microbiome was assessed using the beta-diversity (compositional differences amongst samples) as well as the alpha-diversity (effective number of species in a sample). The beta-diversity measures were calculated using unweighted and weighted UniFrac distances. The alpha-diversity indices were calculated using the Pielou and Shannon diversity.

**Figure 3:**
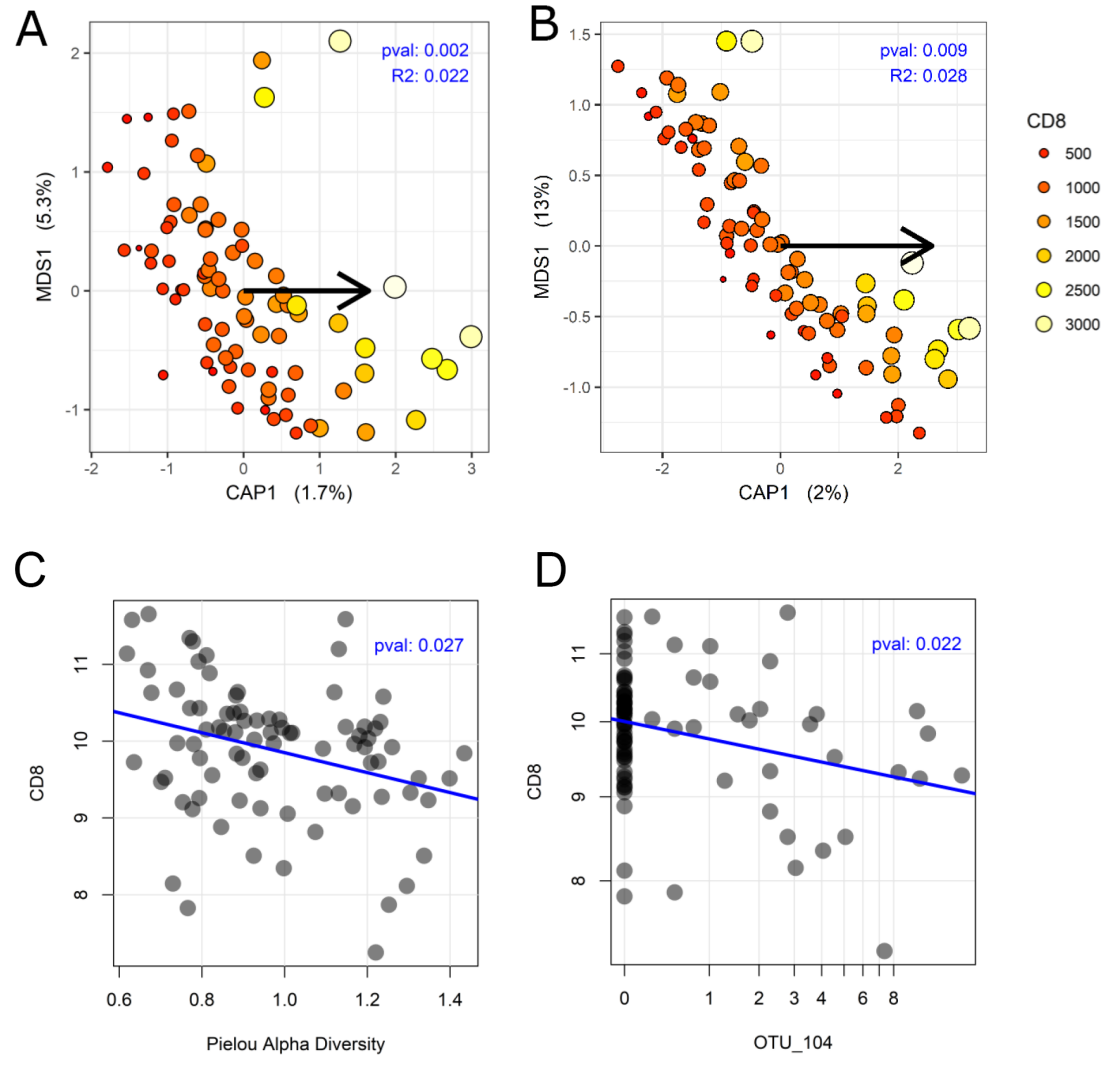
Association between colon cancer microbiome and CD8^+^ **(A)** Capscale ordination using unweighted UniFrac distance. **(B)** Capscale ordination using weighted UniFrac distance. The increased size and increasingly yellow points are the samples whose CD8 values are higher than those of the smaller red points. Each point on the plot represents a sample, whereby a shorter distance between points indicates increasing similarity in bacterial composition. The arrow shows the direction from the origin for which sites have larger abundances for CD8. Adonis test was used to calculate the ‘pval’ and ‘R^2^’ displayed on the ordination plots. **(C)** Scatterplot showing an inverse correlation between CD8 and Pielou alpha diversity. **(D)** Scatterplot showing an inverse correlation between CD8 and the relative abundance of OTU_104. Linear modeling was used to calculate the ‘pval’. The blue line is best fit obtained by linear regression.

CD8^+^ stain showed significant association with the bacterial composition by both unweighted UniFrac (p-value: 0.001) and by weighted UniFrac (p-value: 0.010). To graphically represent the beta-diversity analysis, a capscale ordination was performed using the respective UniFrac distances (Figure 3A, 3B). These data indicate that the CD8^+^ stain is associated with a change in both the species membership and their corresponding abundances. In accordance with the beta-diversity analysis to CD8^+^, the Pielou alpha-diversity showed statistically significant correlation to the CD8^+^ stain (p-value: 0.027) (Figure 3C). There is a decrease in number of unique bacterial species with an increase in CD8^+^ stain. However, the Shannon alpha-diversity did not show a significant correlation to the CD8^+^ stain.

In contrast, the bacterial composition was not associated with the N-stage of the patient. Beta-diversity analysis showed that the bacterial composition between stage III vs. stage I&II was not statistically different by either unweighted UniFrac (p-value: 0.391) or by weighted UniFrac (p-value: 0.112). Alpha-diversity analysis using both the Pielou and Shannon index showed that the effective number of species is not associated with N-Stage (N2 vs N0 p-value: 0.194, N1 vs N0 p-value: 0.780).

### OTU_104 is associated with DFS and CD8^+^ counts

In the univariable Cox regression analysis in Table 1, OTU_104 was associated to DFS with a 1.21 (CI: 1.08, 1.36) times higher chance of recurrence for each percent increase in relative abundance. After applying Benjamini-Hochberg correction, OTU_104 remained statistically significant (p-adjusted: 0.036) (Table 1) out of 36 OTUs tested. Next, we tested whether the relative abundance OTU_104 will also be associated with CD8^+^ quantities. We find that in accordance with their relation to DFS, OTU_104 and CD8^+^ counts have an inversely correlated relationship: with increase in OTU_104, there is a decrease in CD8^+^ counts (p-value: 0.022) (Figure 3D).

In summary, OTU_104, which was identified to the order Clostridiales, is not only associated with a higher risk of colon cancer recurrence, but also a decreased number of CD8^+^ quantity. In effort to narrow the bacterial classification beyond the Order Clostridiales, the consensus V4 sequence for OTU_104 was queried against the NCBI “refseq_rna” using the default setting BLAST algorithm (Basic Local Alignment Search Tool). The sequence mapped with 100% identity to “*Eubacteria rectale* strain (NR_074634.1)” and with 99% identity to “*Roseburia faecis* strain (NR_042832.1).”

### Validation

To validate that the initial findings were not simply due to a rarefaction anomaly or an extreme subsampling of the data, we normalized the same OTU table into proportion by scaling the counts to one. All findings were reproduced except the association between the unweighted UniFrac dissimilarity and the CD8+ quantity. We also tested whether omitting the OTUs present in the negative control samples biased the data to manufacture these findings. When we reanalyzed the data to include those OTUs present in the negative control samples (i.e. using the unsubtracted OTU table), all findings remained consistent (Supplementary Table 2).

## DISCUSSION

Investigation of the complexity and diversity of gut microbiota is critical to understand the normal physiology of gastrointestinal function and pathophysiology of disease. Several extrinsic factors, including alcohol, sugar, over-utilization of antibiotics as well as a diet rich in processed foods likely contribute to the gut microbiota diversity or lack thereof [35, 36]. The mammalian gut is considered a complex ecosystem where the interaction between resident microorganisms and cells regulates the health of the local tissue and the host [37]. Several studies indicate that bacterial dysbiosis may influence colorectal cancer risk [14, 15, 38] and perhaps even prognosis [39–41].

The microbiome has been a huge focus in study for predictive measures in colon cancer. Recently, *Fusobacterium nucleatum* has been described as the main microbe in colon cancer tissue [19, 38–44]. However, others have demonstrated a more diverse pattern of microbes associated with CC in both fecal and tumor tissue human samples [13, 16, 18, 19, 39–42, 45, 46]. In human fecal studies, phylotypes related to genera *Akkermansia* [46, 55], *Bacteroides* [15], *Porphyronomas* [14, 47] and *Parvinomas* [48] were more abundant in CC patients when compared to healthy controls. Analysis of tissue samples showed the presence of a variety of microbiota at genus level: *Prevotella*, *Peptostreptococcus*, *Lactococcus* [18], *Alistipes, Akkermansia, Halomonas, Shewanella, Faecalibacterium, Clostridium* [45], *Providencia* [49] and *Roseburia* [50]. Furthermore, other factors are associated with the composition of the microbiota, such as different sites where cancer (distal, rectal, or proximal) can be found in the gut [51]. In fact, Flemer and colleagues showed a distinct difference between the microbiota found in distal vs. proximal colon cancer tissue [45]. It is unlikely that only one single bacterial species would be responsible, directly or indirectly, for CC development or persistence.

Here, we expanded the investigation of the microbiota of colon cancer tissue to include its association with the local immune microenvironment and DFS. The immune microenvironment has been studied in various types of tumor with prognostic and clinical impact on cancer [29–32, 52, 53]. For this purpose, immunoscore is considered a valuable tool based on the quantification of cytotoxic and memory T cells infiltrating and surrounding the tumor [54, 55]. Studies performed by Galon's group have demonstrated that the tumor-infiltrating immune cells are a more valuable prognostic tool in CC compared to the traditional TNM stage classification [30, 33]. Specifically, T cell immunoscore in CC was shown to be a predictive tool and with more prognostic value than the AJCC staging criteria [30]. In accordance with its predictive values, we had previously found that higher expression of CD8^+^ cells in the tumor center and invasive margin was associated with improved disease free survival (DFS) [34]. Previous research demonstrated significant improvement in overall survival (OS) and DFS in CC patients with high densities of CD8^+^ T cells and increased T cell markers of migration, activation, and differentiation [29, 33]. Our findings are consistent with these studies. Pages et al. showed that high levels of intratumoral memory T cells density are associated with decreased incidence of tumor spread [29] as well as a direct correlation with clinical outcome, providing biomarkers for tumor recurrence and patient survival [33].

In our sample cohort taken from the only prospective clinic trial evaluating staging in colon cancer with attention to both surgical and quality standards, we found high levels of infiltrating T cells linked to better patient prognosis [33, 56], consistent with other reports [57]. We found that one OTU, OTU_104, was associated with a poorer DFS even after appropriately correcting for multiple hypothesis testing. We were surprised to find that this OTU was also associated with a lower CD8^+^ level. Regarding CC prognosis, high abundance of *F. nucleatum* in tumor tissue was associated with poor survival, possibly serving as a prognostic biomarker [48, 49] along with the presence of *Bacteroides fragilis* [17]. *Faecalibacterium prausnizii* and *Methylobacterium* were found with higher abundance in the group with better survival [50]. Our study did not detect any differences in *F. nucleatum* with DFS. In contrast, our model indicates that DFS is associated with N-Stage, CD8^+^ stain, and OTU_104. After correcting for multiple hypothesis testing, only OTU_104, which matches with *Eubacteria rectale* (100% identity) and *Roseburia faecis* (99% identity), is associated with a decreased DFS. Additionally, this OTU_104 was found to be inversely correlated to CD8^+^ count.

We further validated our findings using two additional approaches to ensure our results were not a rarefaction anomaly or an artifact from subtracting OTUs present in the negative controls. Using the data normalized by scaling the counts into proportions, or using the raw data without excluding OTUs present in the negative controls, we found the same results with the exception that the association between CD8+ and the unweighted UniFrac was no longer statistically significant. This is expected, as normalization by proportion rather than rarefaction would include all rare-occurring OTUs with very low abundance and bias the unweighted beta-diversity based on the number of reads in the sample.

*E. rectale/Roseburia,* similar in sequence [58, 59], have been described to produce short-chain fatty acids (SCFAs) where acetate, propionate and butyrate are the main fermentation end products from the indigestible dietary fiber [58, 60]. Of these, butyrate has been intensely studied. However, its role in CC progression [61, 62] or prevention [63–66] is controversial. Furthermore, butyrate interferes with immune cell functions and cytokine modulation in response to several stimuli [67–70]. In fact, in an inflammatory environment, butyrate might act to suppress inflammation by inhibiting IFN-gamma induced STAT1 activation, which in turn would inhibit iNOS upregulation [67, 71, 72] and downregulate B7-1 (CD80), ICAM-1 (CD54), and LFA-3 (CD58) expression on monocytes to alter APC function [73]. All of these could lead to inhibition of T lymphocyte proliferation [67, 71] and apoptosis of both CD4^+^ and CD8^+^ T cells [67, 70, 74]. Therefore, butyrate may affect host immune function, which might affect an anti-tumor response.

Furthermore, while some have shown butyrate is associated with apoptosis of cancer cells [63, 64], in high concentrations of butyrate, tumor cells acquire the capacity that normal cells have to metabolize butyrate [61, 62]; thereby, cancer cells were able to avoid the HDAC inhibition [75] and become more malignant and aggressive [61]. Recently, a study using an APC^min/+^ mice (multiple intestinal neoplasia) showed that microbes play a role on CC by boosting the hyperproliferation of cancer cells through metabolites such as butyrate [76]. Lastly, while ingestion of fiber (the main source for fermentation to SCFAs) has been associated with beneficial effects for overall health [77, 78], other studies have shown that fiber consumption may be not beneficial to prevent colorectal adenoma recurrence (a precursor for CC) [79–81]. Therefore, the role for fiber and SCFA in CC is unclear.

Given that OTU_104 (*E.rectale/roseburia*) in the colon cancer tissue was associated with both a higher risk of recurrence and lower CD8^+^ levels, our result suggests an association of microbiota with CD8^+^ cells in the tumor tissue. In this regard, it is tempting to speculate that *E.rectale*/*Roseburia* might have an impact in the tumor development. Future investigations are required to further validate these findings in additional cohorts and also to elucidate causality of *E.rectale/Roseburia* and SCFAs with clinical outcomes and recurrence in CC; however, these are beyond the scope of this study.

## MATERIALS AND METHODS

### Study population

A cohort of 91 patients, 31 from California and 54 from Serbia, was randomly selected from patients enrolled in an ongoing prospective multicenter trial of nodal ultra-staging in early stage colon cancer using pathological and surgical quality standards (NCT0094932). All experimental protocols were approved by the Western Institutional Review Board (protocol number 20120978) and specimens received in the Lee laboratory from Department of Surgical Oncology - John Wayne Cancer Institute at Providence St. John's Health Center, Santa Monica, CA, USA were de-identified and accepted under an IRB exemption approved by John Wayne Cancer Institute Regulatory affairs.

Written informed consent was obtained from all subjects. All experiments involving the use of human tissue samples were performed in accordance with the Common Rule (45 CFR 46), ICH E6 GCP guidance as well as the Western IRB's requirements for consenting subjects.

### Preparation of samples for 16S rRNA gene amplicon sequencing

Second Genome performed nucleic isolation from formalin-fixed paraffin embedded (FFPE) colon cancer blocks obtained during surgical excision of the primary tumors as well as from paraffin shavings (without tissue) with the MoBio PowerMag® Microbiome kit (Carlsbad, CA, USA) according to manufacturer's guidelines and optimized for high-throughput processing. All samples were quantified via the Qubit® Quant-iT dsDNA High Sensitivity Kit (Invitrogen, Life Technologies, Grand Island, NY, USA) to ensure that they met minimum concentration and mass of DNA.

DNA was sequenced by 16S rRNA gene amplicon sequencing by Second Genome (Second Genome, The Microbiome Company, San Francisco, USA) including no template controls (NTC). The 16S rRNA gene amplicon - V4 region was enriched, amplified, and paired-end sequenced for 250 cycles on the MiSeq instrument (Illumina MiSeq). The DNA from each sample was amplified using Caporaso primers tailed with sequences to incorporate flow cell adapters and indexing barcodes [82].

### Sequence processing

The Illumina MiSeq generated a total of 22 million, 251 base-pair (bp), paired-end reads, that were joined using VSEARCH with a minimum overlap of 200 bp and maximum differences of 30 bp. Joined sequences were trimmed to 251 bp and filtered to a maximum expected error of 1, resulting in 15.9 million high-quality sequences. Reads were pooled, de-replicated and chimera checked with UCHIME de novo as implemented in VSEARCH [83], followed by UCHIME using the RDP Gold database [84]. Remaining sequences were clustered into OTUs at 97% similarity. A feature-abundance table was constructed by matching all high-quality reads to these centroids. Taxonomy was assigned to each OTU centroid using the May 2013 version of the Greengenes database and a last common ancestor approach as implemented in QIIME v1.9.1 [85]. Centroids were then aligned to Greengenes and a phylogenetic tree was constructed using FastTree2 [86, 87]. OTUs with >5% relative abundance in no template controls (NTC), paraffin shavings, and empty Eppendorf tubes were excluded from the analysis. Additionally, any non-singleton OTUs found in the NTC were excluded as well. After subtracting the background noise in the environmental controls, the remaining samples were rarefied to a sequencing depth of 394 reads.

### Statistical analysis

The two beta-diversity measures, unweighted UniFrac and weighted UniFrac, were calculated using the rarefied OTU table and a phylogenetic tree. The unweighted UniFrac reflects differences in community membership such as the presence and absence of an OTU. The weighted UniFrac, on the other hand, additionally captures the differences in abundance. Permutational multivariate analysis of variance (PERMANOVA), also known as Adonis test, was performed to determine whether the samples clustered by their beta diversity partition distance. Plots for beta-diversity analysis were constructed by capscale ordination, in which each point on the plot is a sample, and the shorter distances between points indicate increasing compositional similarity. The alpha-diversity (effective number of species) was calculated using five different measures: Shannon, Pielou, SimpsonD, SimponE, and raw observed number of OTUs (species richness).

Prior to performing Cox regression, alpha diversity values were log2 transformed and the OTU table was scaled to 1 so that the resulting hazard ratios are interpretable. As an additional filter, only OTUs present in at least 10% of the samples from California as well as in at least 10% of the samples from Serbia were included. This ensures that we did not study extremely rare and location specific OTUs. The Benjamini-Hochberg correction was applied to account for false discovery associated with multiple hypothesis testing.

Cox proportional hazard regression was used to model the association of categorical and quantitative variables to DFS. Prior to MVA, a univariable cox regression screened for candidate predictor variables with an alpha threshold of 0.20 (Supplementary Table 1). The final model included N-Stage, CD8^+^, and OTU_104 as a function of DFS. The final model was resolved by a series of ANOVA tests comparing the deviance between possible Cox models. The final model does not violate the proportionality assumption, which was tested by a Pearson correlation between the scaled Schoenfeld residuals and log(time) for each covariate. Kaplan–Meier plots were included for easier visualization and interpretation for both categorical and continuous variables. The threshold to dichotomize a continuous variable into two groups was at the population mean.

Linear regression modeling was used to assess the relationship between variables. When relevant, statistical models included the “geographic location” as a covariate to account for the batch effect between the two cohorts. All statistical analyses and figures were generated in R 3.3.1 with the help of Phyloseq [88] and vegan package [89].

### Validation

The findings were validated by two methods. In the first, the OTU table was normalized by scaling the sample counts into 1 (into proportions) and only including OTUs with > 0.01% relative abundance in at least one sample to test whether the finding is due to extreme subsampling of the data. To test whether the finding was influenced or biased by the subtraction of OTUs in the negative controls, the data was also analyzed without omitting OTUs found in the environmental controls and rarefied to a sampling depth of 1011 reads.

## CONCLUSIONS

Changes in the microbiota during the carcinogenesis process still remains unclear, and most importantly, how far gut dysbiosis could contribute to the development of colon cancer or its prognosis requires further study. Our study is the first to evaluate levels of CD8^+^ T cells in association with the colon cancer tissue microbiome and DFS in a prospective clinical trial, the only prospective clinic trial evaluating staging in colon cancer with attention to both surgical and quality standards. Further studies are warranted to investigate the role of specific microbes, their role in influencing outcomes in colon cancer patients, and whether there is a direct or indirect role on the host anti-tumor response.

## SUPPLEMENTARY MATERIALS FIGURES AND TABLES



## Abbreviations

CC: colon cancer
DFS: Disease-free Survival
OTUs: Operational Taxonomic Units
MVA: Multivariable Analysis
HR: Hazard Ratio
CI: Confidence Interval
IRB: Institutional Review Board
CFR: Code of Federal Regulation
ICH E6 GCP: Interna tional Council document E6 Good Clinical Practice
IHC: Immunohistochemistry
FFPE: Formalin-fixe Paraffin-embedded
DNA: Deoxyribonucleic acid
RNA: Ribonucleic acid
NTC: No template controls
SCFAs: Short-chain Fatty acids
HDAC: Histone Deacetylase
GPCRs: G-protein couple receptors
CD4: cluster of differentiation 4
CD8: cluster of differentiation 8
CD80: cluster of differentiation 80
CD54: cluster of differentiation 54
LFA-3: Lymphocyte function associated-3
CD58: cluster of differentiation 58
IL-10: Interleukin 10
Il-17: Interleukin 17
IFN-γ: Interferon-gamma
iNOS: inducible Nitric Oxide Synthase
STAT1: Signal transducer and activator of transcription 1
ICAM-1: Intercellular adhesion molecule-1
atRA: all-trans-retinoic acid
