# Supplementary material for: Both the intratumoral immune and microbial microenvironment are linked to recurrence in human colon cancer: results from a prospective, multicenter nodal ultrastaging trial
Source: Oncotarget. 2018 May 4;9(34):23564–76. doi: 10.18632/oncotarget.25276 (PMC5955112; doi:10.18632/oncotarget.25276)
Supplement: Supplementary file 1 [file oncotarget-09-23564-s001.pdf]

# Both the intratumoral immune and microbial microenvironment are linked to recurrence in human colon cancer: results from a prospective, multicenter nodal ultrastaging trial

SUPPLEMENTARY MATERIALS

Supplementary Table 1: Clinical information and univariable cox regression

	Summary	Hazard Ratio (95% CI)	P-Value
**Clinical Parameters**			
**Geographical Location**	California = 31	1	---
	Serbia = 54	1.61 (0.58, 4.44)	0.360
**Sex**	Female = 39	1	---
	Male = 46	0.68 (0.26, 1.77)	0.431
**Age**	67.0 ± 12.6	0.97 (0.93, 1.00)	0.079
**BMI**	25.9 ± 4.4	1.03 (0.92, 1.15)	0.649
**Tumor Size**	4.48 ± 1.9	0.93 (0.71, 1.21)	0.577
**Stage**	I & II = 37	1	---
	III = 48	3.67 (1.05, 12.81)	0.041
**N.Stage (Lymph nodes involved)**	N0 = 37	1	---
	N1 = 40	3.31 (0.92, 11.92)	0.067
	N2 = 8	5.94 (1.19, 29.60)	0.030
**Positive Lymph Nodes**	Negative = 37	1	---
	Positive = 48	1.39 (1.06, 1.81)	0.017
**Microsatellite Instability**	Negative = 65		
	Positive = 13	1	---
	Unknown = 7	0.34 (0.04, 2.61)	0.300
**Lymphovascular Invasion**	Negative = 57	1	---
	Positive = 28	1.61 (0.55, 4.68)	0.381
**Colon Side**	Left = 41	1	---
	Right = 36	0.61 (0.22, 1.68)	0.334
	Other = 8	0.58 (0.07, 4.57)	0.605
**Immune Markers (Histology)**			
**CD3**	10.8 ± 0.79	0.58 (0.37, 0.91)	0.017
**CD4**	10.4 ± 0.85	0.66 (0.42, 1.04)	0.071
**CD8**	9.9 ± 0.87	0.39 (0.23, 0.66)	< 0.001
**CD68**	11.3 ± 0.53	0.79 (0.17, 3.71)	0.769
**FOXP3**	8.1 ± 2.31	1.07 (0.82, 1.40)	0.596
**Microbiome (16S-V4)**			
**Shannon α-Diversity**	0.98 ± 0.19	2.65 (0.18, 38.76)	0.477
**Pielou α-Diversity**	0.97 ± 0.21	4.31 (0.42, 44.49)	0.220
**SimpsonD α-Diversity**	0.94 ± 0.32	1.88 (0.44, 8.04)	0.396
**SimpsonE α-Diversity**	0.79 ± 0.61	1.46 (0.79, 2.72)	0.228
**Observed OTU**	36.0 ± 21.40	1.00 (0.97, 1.02)	0.728

Summary of continuous variables shown as 'mean ± standard deviation'
Univariable Cox regression was performed to screen for predictor variables related to tumor recurrence rate.
Variables meeting alpha threshold 0.20 (80% level of confidence) are taken into consideration in the multivariable analysis.

Supplementary Table 2: Validation

	Controls Subtracted Rarefied (Reported Results)	Controls Subtracted Proportioned (Validation)	Not Subtracted Rarefied (Validation)
DFS & $CD8^+$	***	***	***
DFS & OTU104	***	***	***
DFS & Shannon Alpha Diversity	NS	NS	NS
DFS & Pielou Alpha Diversity	NS	NS	NS
DFS & observed number of OTUs	NS	NS	NS
Unweighted UniFrac & $CD8^+$	**	NS	*
Weighted UniFrac & $CD8^+$	*	*	*
$CD8^+$ & OTU104	*	*	*
$CD8^+$ & Shannon Alpha Diversity	NS	NS	NS
$CD8^+$ & Pielou Alpha Diversity	*	*	*
$CD8^+$ & observed number of OTUs	NS	NS	NS

***: < 0.001
**: < 0.01
*: < 0.05
NS: Not Significant
The data was normalized by two independent methods and reanalyzed to determine whether the reported results were consistent.The level of significance is indicated by the number of stars.
